# rAAV Manufacturing: The Challenges of Soft Sensing during Upstream Processing

**DOI:** 10.3390/bioengineering10020229

**Published:** 2023-02-08

**Authors:** Cristovão Freitas Iglesias, Milica Ristovski, Miodrag Bolic, Miroslava Cuperlovic-Culf

**Affiliations:** 1 Faculty of Engineering, University of Ottawa, Ottawa, ON K1N 6N5, Canada; 2 Faculty of Medicine, University of Ottawa, Ottawa, ON K1H 8M5, Canada; 3 Digital Technologies Research Center, National Research Council, Ottawa, ON K1A 0R6, Canada; 4 Department of Biochemistry, Microbiology, and Immunology, Faculty of Medicine, University of Ottawa, Ottawa, ON K1H 8M5, Canada

**Keywords:** recombinant adeno-associated virus, rAAV production, bioprocess optimization, soft sensing

## Abstract

Recombinant adeno-associated virus (rAAV) is the most effective viral vector technology for directly translating the genomic revolution into medicinal therapies. However, the manufacturing of rAAV viral vectors remains challenging in the upstream processing with low rAAV yield in large-scale production and high cost, limiting the generalization of rAAV-based treatments. This situation can be improved by real-time monitoring of critical process parameters (CPP) that affect critical quality attributes (CQA). To achieve this aim, soft sensing combined with predictive modeling is an important strategy that can be used for optimizing the upstream process of rAAV production by monitoring critical process variables in real time. However, the development of soft sensors for rAAV production as a fast and low-cost monitoring approach is not an easy task. This review article describes four challenges and critically discusses the possible solutions that can enable the application of soft sensors for rAAV production monitoring. The challenges from a data scientist’s perspective are (i) a predictor variable (soft-sensor inputs) set without AAV viral titer, (ii) multi-step forecasting, (iii) multiple process phases, and (iv) soft-sensor development composed of the mechanistic model.

## 1. Introduction

Recombinant adeno-associated viruses (rAAV) are a class of viral vectors engineered to express specific genes or therapeutic proteins in cells [1,2,3]. They are used in gene therapy as vectors to deliver genetic material to specific cells with high efficiency and low toxicity [1,4,5]. rAAVs are attractive vectors for gene therapy due to their ability to integrate their genetic load into a specific location in the host genome, remain a silent passenger in the host genome, and not cause disease [5,6,7,8]. Currently, rAAVs are being evaluated in preclinical and clinical trials for a wide range of genetic diseases [3,6,9,10], including inherited retinal diseases [11,12], muscular dystrophies [13,14,15,16], and hemophilia [17,18]. rAAVs have also shown promise in gene therapies for cancer [19], cardiovascular diseases [20,21], and neurological disorders [22,23]. The most common method for rAAV production is through transfection of host cells, such as HEK293 cells, with plasmids encoding for rAAV Rep and Cap proteins, and the therapeutic gene of interest [18,24,25]. However, for rAAVs to be used effectively in gene therapy, large amounts of high-quality virus particles are required [14,26,27]. Therefore, the production of high-quality virus particles is critical for the advancement of rAAV-based therapies.

The current state of rAAV research is focused on developing new methods to produce high-quality virus particles in large amounts [2,28,29,30,31,32,33,34,35]. Researchers have been developing new methods such as serum-free suspension systems [10,36], stable cell lines [37,38], helper-dependent AAV systems [39], high-throughput systems [40], and other methods such as using baculovirus-insect cells systems [41]. Furthermore, Srivastava et al. [42] describe the numerous manufacturing processes and difficulties faced during the manufacture and storage of rAAV and provide a road map for enhancing manufacturing workflow efficiency and extending product shelf-life. Dobrowsky et al. [43] present an overview of the manufacturing-related obstacles resulting from using this vector for clinical purposes, mainly focusing on the difficulties associated with the upstream process. However, these studies do not comment on soft sensing as a mitigation strategy to improve the scalability, reproducibility, and cost-effectiveness of rAAV production, which is crucial for commercializing rAAV-based therapies. For example, currently, a typical production run of an rAAV-vector treatment utilizing high-yield cell lines and large-capacity bioreactors may only produce ten doses of a systemic gene therapy from a single batch at the cost of about USD 100,000 per dosage (considering around 1 × $10^{17}$ vg per batch) [44]. Consequently, any technological advancement that decreases the cost per dose would be immediately advantageous [44]. Soft sensors have been used as a fast and low-cost approach to control and monitor the bioprocesses by enabling real-time screening and control of critical process variables of the upstream process to ensure product quality and process consistency [45,46,47,48,49,50]. Furthermore, this approach is a recommendation of the Food and Drug Administration’s process analytical technology initiative (PAT). They suggest the continuous in-process monitoring of critical process variables to assure product quality at every stage of the manufacturing process [51].

The literature presentation of the usage of soft sensors for rAAV production is limited. Recently, two review articles described approaches of relevance in soft sensors in rAAV production. Tsopanoglou et al. [52] discuss the advantages and challenges of hybrid modeling for upstream pharmaceutical bioprocesses, and Joiner et al. [53] proposed an outline (high-level framework) for the future development of a hybrid mechanistic and data-driven model for rAAV production modeling. However, the main challenges of soft-sensing in rAAV production, such as the design and applications were, best to our knowledge, not considered.

Therefore, to encourage and provide a background necessary for the advancement of the field, this review article discusses the advantages and challenges of applying soft sensors as a fast and low-cost methodology for rAAV production monitoring based on current knowledge of rAAV biology and manufacturing. Furthermore, we present the possible solutions to side-step these challenges and a glossary (Table S1 in the Supplementary Material) provides common vocabulary bridging between data scientists and biologists. The challenges from a data scientist’s perspective are: (i) a predictor variable (soft sensor inputs) set without rAAV viral titer, (ii) multi-step forecasting, (iii) multiple process phases, and (iv) soft sensor development composed of the mechanistic model.

In this review, we discuss the advantages of soft-sensing in the upstream process of rAAV production in Section 2. We further discuss the soft sensor design in Section 3. The following Section 4 is concerned with the four challenges and possible solutions in soft sensor development for rAAV production from a data scientist’s perspective. Section 5 concludes with a final discussion and a summary of the topic.

## 2. Soft Sensor in Upstream of rAAV Production

Viral vector manufacturing is a complex process that involves an upstream and downstream component and takes into account the maintenance of the viral vector’s long-term stability and efficacy. The upstream process is comprised of four steps: plasmid development [54,55], cell expansion [56], plasmid transfection [8,57], and viral vector production [25,42]. The challenges of the upstream process of rAAV production are the need to increase the viral titer and decrease the variability in product quality [42]. These challenges in rAAV manufacturing resemble those that monoclonal antibody manufacturers faced [42]. From a biological viewpoint, two approaches can be used: (i) developing stable rAAV producer suspension cell lines that do not require triple transfection or (ii) utilizing other innovative technologies that are plasmid-free [42]. There is active development in both areas, but these are long-term solutions requiring more investigation and investment. On the other hand, mathematical modeling is a possible immediate solution from the biomanufacturing monitoring and control viewpoint since it minimizes the production time and cost while also learning about the critical process steps [52,58,59]. Mathematical models are increasingly being used in the biomanufacturing industry to design and optimize different processes and unit operations [59,60]. Mathematical modeling that can optimize the upstream process is based on the concept of soft sensors. Soft sensors use physical or machine learning models that predict or estimate quantities of interest based on raw data [61,62]. They consist of mathematical models that use data from a measurement device (input) to predict a quantity of interest (output), which is used to generate new information about the bioprocess [50,63].

Biopharma 4.0 is the next step in the digitalization of biopharmaceutical manufacturing that aims to optimize biopharmaceutical production efficiency by minimizing human intervention and the uncertainty that arises from it [52]. Soft sensors are a crucial enabler of Biopharma 4.0 since it enables real-time monitoring of variables that are difficult to measure directly or that can only be measured at a low sampling frequency [52,64]. This is a vital step towards the digital transformation of bioprocesses [52]. The application of soft sensors in upstream rAAV production can contribute to the reduction of time and production costs, the generation of relevant data, improved control of bioprocesses, increased product quality, and the reduction of raw material waste [50]. The large potential of mathematical models used in soft sensors lies in the possibility of establishing quantitative links between critical process parameters (process variables that have an impact on a critical quality attribute) and Key Performance Indicator (KPI) providing data for bioprocess optimization [50,52,65,66]. Furthermore, soft sensors are used as a base of advanced control strategies for bioprocess optimization, such as digital twins [59,67,68], model predictive control [69,70], and model-based control [45].

Soft sensors have been used to control and monitor the production of mAb in Chinese hamster ovary (CHO) cell cultures, and enable the real-time screening and control of critical process variables of the upstream process to ensure product quality and process consistency [71,72,73,74,74]. Besides applying soft sensors in mAb production, they were also developed for a number of other bioprocess applications. For example, Zhang et al. [75] used a soft sensor based on artificial neural networks to monitor the fermentation process by measuring glycerol, 1,2 propanediol, and biomass. Chromatography monitoring was performed with a soft sensor based on partial least squares regression with the help of mid-UV absorption spectra [76] as well as Fourier transform mid-infrared spectroscopy [77]. Several papers used soft sensors to estimate the biomass and substrate concentrations [78,79,80,81,82]. Krishna et al. [83] calculated the lactose and ethanol concentrations with deep learning-based soft sensor models.

Since soft sensors have helped improve quality and production rates for mAbs [71,72,73,74,74], one can expect that a similar methodology would be beneficial for rAAV production. rAAV production utilizes a mammalian cell culture-based process similar to mAb production and faces a similar upstream challenge as was faced by mAb production [42,52,84]. Therefore, soft sensors have a high potential to provide insight into the underlying micro and macro-scale phenomena of upstream bioprocesses of rAAV manufacturing and improve the yield and quality of production [52].

## 3. Designing Soft Sensors

Soft sensor design should combine analytical and computational methods with adequate mathematical models for efficient modeling and accurate predictions of bioprocesses. Three kinds of modeling approaches are commonly used: the Data-Driven Model (DDM), the Mechanistic Model (MM), and the Hybrid Model (HM), which aims to combine the advantages of DDMs and MMs [50].

### 3.1. Data Driven Models

DDMs are built purely on empirical observations of a process [50]. They establish links between input and output variables (system state variables) without having explicit knowledge of the physical laws in the system [50,85]. DDMs are mainly based on machine learning techniques that may be used to create models that can either supplement or completely replace physics-based models. DDMs learn the link between the inputs and outputs of a system from a training data set, and after the DDM has been trained, its ability to generalize to new data may be assessed using an independent data set [50,85]. Some of the most common data-driven modeling techniques used in related applications are: fuzzy rule-based systems [85], Partial Least Squares (PLS) [76,77], Artificial Neural Networks [75], Support Vector Machine (SVM) [85], and Deep Learning (DL) [86]. In the last two decades, DL models composed of multiple hidden layers have been shown to be capable of learning data representations with various levels of abstraction [86]. As a result, these methods have dramatically improved many domains, such as drug discovery and genomics [87]. Research that uses DL methods in soft sensors has been gaining popularity in recent years [86]. The advantages of DL approaches over conventional algorithms mainly result from (i) learning relevant process behaviors without knowledge or experience about process mechanisms and (ii) taking full advantage of the massive amount of data available in public data sets for performance improvements [49,86,88]. Typical models, i.e., architecture, in the DL field for soft sensors include Autoendocoder (AE), Convolutional Neural Networks (CNN), and Recurrent Neural Networks (RNN), such as Long Short Term Memory (LSTM) networks [86].

**DDM Advantages.** DDMs, compared to kinetic models, have more parameters and structures for data regression, allowing the integration of various process behaviors obtained under different operating circumstances in a single model. This is done using a training set that can represent all possible behaviors seen in the system [89]. In addition, DDMs can identify patterns and trends in complex and heterogeneous data, thus facilitating informed decisions and actions without insight into the fundamental mechanism of the process [52,90]. Another important advantage of DDMs is that the development time is shorter than that of MM design [52].

**DDM Disadvantages.** Unfortunately, DDMs have several significant disadvantages. For example, complex DDMs, such as deep learning models, perform predictions without consideration of the underlying mechanisms, resulting in parameters with no physical, chemical, or biological meaning. The variables are based on correlations and not necessarily on causation [52]. Furthermore, poor data quality and low data quantity are a major concern for DDMs. The variables can only be predicted with high accuracy in the space adequately represented by the training set. This means that generalization ability relies on training with large amounts of data [52,90]. Although designing DDMs is usually less time-consuming than designing an MM [52,91], DDMs may require pre-processing steps, which can be time-consuming [52]. DDMs need accessible, representative, and reliable training data sets. DDMs can have limited bioprocess control and optimization capabilities and frequent model drift (the degradation of the prediction power of the model due to the changes in the statistical properties of the input features, target variable, or relationships among variables). Re-calibration is often time-consuming [50] and data demanding.

**DDM in upstream rAAV production.** DDMs are often applied in academia and industry, particularly in the chemical process sectors [50]. An initial study using DDMs for the upstream processing of rAAVs was recently published. Iglesias et al. [92] developed an approach for handling a massive proportion of missing labels in multivariate, multi-step time series forecasting. The approach is a two-step process where interpolation (using Gaussian Processes Regression (GPR) and domain knowledge from experts) and prediction modeling are separated to integrate the prior domain knowledge. The authors evaluated their approach by developing a conceptual soft sensor to forecast the biomass of HEK293 cells in rAAV production. The soft sensor was designed using Long Short-Term Memory encoder-decoder architecture to forecast the biomass in the next 1 h based on measuring the last hour of three input features representing the model’s input. The input features include: (i) Cumulative O2 (mL), (ii) Dissolved Oxygen (%), and (iii) Cumulative Dissolved Oxygen. The model was trained and tested with a real data set obtained during rAAV production performed in 3L bioreactors [92].

### 3.2. Mechanistic Models

Physical, chemical, and biological insights and understanding of the transformation processes in producing organisms are used in MMs development. The recommended mechanistic technique for capturing the underlying events during cell culture is kinetic modeling [50,52,93]. Mechanistic, white-box or parametric models are considered a priori, based on the knowledge of the process. The number of model parameters is fixed, and they might have a physical or empirical interpretation depending on the level of knowledge.

Usually, MMs are represented as systems of ordinary or partial differential equations, and the numerical values are assigned to the MMs parameters via parameters estimation [94,95,96,97,98] (see Section S1 in the Supplementary Materials). Two approaches that can be used are Neural Ordinary Differential Equation (NODE) [99,100,101,102,103,104,105,106] and Bayesian Inference [107,108] (see Section S1 in the Supplementary Materials). Furthermore, MMs can be categorized as structured and unstructured models [52,93].

Unstructured models treat all cells as black boxes, assuming they are all in the same physiological condition. Extracellular factors are the only ones that impact cell growth and behavior. Unstructured models rely on nutrient and metabolite concentrations in cultures while ignoring intracellular activities. In mammalian cell cultures, unstructured kinetic models have also been utilized to estimate recombinant protein output and can be used in the upstream rAAV production [93,109]. The Monod model, which assumes that the concentration of a substrate is the growth limiting factor, is a commonly used unstructured model [93]. For example, Kornecki et al. [84] used a Monod-type process model for the simulation of dynamic cellular states (i.e., lag, exponential, stationary, decline phase), as well as the uptake of substrates (i.e., glucose, glutamine), production of metabolites (i.e., lactate, ammonium), and the product (i.e., monoclonal antibody). Because of the dynamic behavior of the culture, yield coefficients can be quantitatively defined by systematically identifying cellular phases (i.e., lag, exponential, stationary, decrease). In addition, cell-dependent half-maximum rates influence the growth rate, and the model parameters were based on maximal growth rates at various substrate concentrations. They were not susceptible to change during culture.

A structured model expresses intracellular activities in structural and physiological terms, providing the most accurate picture of a cell [93,109]. Biomass is divided into sections with diverse functions in structured models. As a result, cells are not considered homogeneous, increasing the complexity of the models and the number of model parameters. It is essential to point out that, although changes in cell attributes may be described, it is very commonly assumed that all cells are the same and change at the same rate. In addition, most kinetic models in the literature are unstructured, and a structured model is not appropriate for most cell lines [93,109].

Structured and unstructured models are further divided into segregated and non-segregated models. Segregated models take into account cell populations when not all cells are homogeneous, non-segregated models consider that all cells are in an identical state [52,93]. Segregation refers to cell population heterogeneity. Similarly to structure, if the model is non-segregated, it does not have this information, and if it is segregated, it has the information [52]. Segregated models are more complex than non-segregated models. Their complexity makes parameter identification and the utilization of optimization algorithms challenging and time-consuming [93].

**MM Advantages.** The advantage of MMs is that they aim to represent process knowledge and generally show a higher extrapolation power than DDMs, leading to more applications. Once they are developed, MMs are reliable and reproducible, offering accurate and relevant information about the process. MMs are based on mechanistic knowledge in biology, chemistry, and physics and yield enhanced process understanding. MMs are an excellent candidate for optimization and can be used for model-based design of optimally informative experiments.

**MM Disadvantages.** The development of MMs may take longer than that of DDMs. The MMs require expertise in the application domain. If an MM is too simple, it may have limited extrapolation power [91]. On the other hand, if there are too many parameters, then the model is hard to train and may lack robustness and universality [52]. Although MMs seem to provide the most accurate and in-depth information, the main disadvantage is that extensive experimental effort is required for model validation. Due to the requirement for in-depth knowledge of the process, it may not be easy to automate and formalize model assembly. Finally, MMs may be difficult to deploy within the industry because of the high level of expertise required for operation and calibration [50]. When there is a limited understanding of underlying intracellular mechanisms and regulation between process inputs and outputs in mammalian cell cultures, the calibration of an MM is a difficult task [94,95].

**MM in upstream rAAV production.** We are at the beginning of the mathematical modelling for rAAV production. Nguyen et al. [110] presented the first structured MM for rAAV production. The authors proposed a mechanistic model for synthesizing rAAV viral vectors by triple plasmid transfection based on the underlying biological processes gathered from wild-type rAAV to better understand the dynamics of recombinant viral generation. The model includes essential phases such as exogenous DNA delivery, the reaction cascade that produces viral proteins and DNA, resulting in filled capsids, and the Rep protein’s complex activities as a regulator of packing plasmid gene expression and a viral DNA packaging catalyst. This model has demonstrated that it can provide valuable insights into experimental data, point out process bottlenecks, and direct future studies. On the other hand, recently, Iglesias et al. [111] presented the first Extended Kalman Filter (EKF) that uses an unstructured mechanistic model as a process model to monitor rAAV production. The proposed approach uses only online viable cell density (Xv) measurements to estimate the other process state variables, including the concentrations of glucose (GLC), glutamine (GLN), lactate (LAC), ammonium (AMM), and rAAV viral titers measured at a low sampling frequency.

### 3.3. Hybrid Models

Hybrid or grey-box models combine nonparametric and parametric models that are based on different types of knowledge [112,113,114]. Furthermore, the vast majority of hybrid modeling studies explored the combination of MM and DDM [115]. The information available through mechanistic modeling is the a priori knowledge in the form of material and energy balances, thermodynamics, and kinetic laws. In addition, mechanistic relationships, such as physical, chemical, or biochemical kinetics, are covered by mechanistic models [93]. Data-driven knowledge, in the form of heuristics, is the second type of knowledge. For example, heuristic process knowledge (e.g., thresholds for inhibition, limitation or optimal temperature, pH, DO ranges) is frequently expressed in fuzzy sets, and historical data are transformed into models by neural networks [93].

Hybrid modeling compromises the benefits and drawbacks of MMs and DDMs. Tsopanoglou et al. [52] state that HMs may lead to safer, more effective, personal, and low-cost products. While MMs can provide predicted outcomes, their construction is time-consuming and needs comprehensive process understanding. DDMs, on the other hand, are easy to construct and use, but they only have acceptable descriptive features within the range of the data they are based on. Compared to solely data-driven modeling techniques, the major benefit of hybrid models is that they can achieve higher accuracy, more efficient model creation, and better extrapolation features [93]. The first challenge in developing HM is figuring out the right way to combine MM and DDM for a specific application, and this can be done in parallel or sequential (cascade) ways [112,116]. In addition, one disadvantage of HM is that the implementation and their parameterization algorithms are highly prone to errors and are time-consuming [112,116].

**HM in upstream rAAV production.** Hybrid models have been successfully applied in bioprocess control, monitoring, and optimization in bioreactors. Therefore, they could be utilized to design soft sensors [50,93] but, so far, no HM has been developed and reported to rAAV production monitoring in the upstream process.

## 4. Challenge of Soft Sensors in Upstream rAAV Production and Possible Solutions

Since soft sensors are sometimes the only way to determine critical process parameters (CPP) or critical quality attributes (CQA) online, they have grown in importance within quality by design (QbD) and process analytical technology initiatives. However, data scientists face several significant challenges in developing soft sensors for rAAV production: (i) Predictor variable (soft sensor inputs) set without rAAV viral titer, (ii) Multi-step forecasting, (iii) Multiple process phases, and (iv) soft sensor development composed of MMs.

### 4.1. Predictor Variables (Soft Sensor Inputs) Set without rAAV Viral Titer

The current techniques for monitoring rAAV manufacturing in bioreactors are expensive, laborious, and time-consuming. The measure of the CPPs, such as cell density and metabolites, and the quantification of the CQAs, such as rAAV genome titer by quantitative polymer chain reaction (qPCR)/Droplet Digital polymerase chain reaction (ddPCR) or viral capsid titer by Enzyme-linked immunosorbent assay (ELISA), takes one day to complete and requires sampling [117]. Recently, in situ monitoring technologies, such as Raman spectroscopy [118,119] and fluorescence spectroscopy [120,121,122], have been developed to estimate the cell density and measure metabolite concentrations in mammalian cell cultures in real-time but there were no reports thus-far on using them to estimate rAAV titer. Additionally, the setup of a spectroscopic system is expensive in terms of both financial outlay and calibration work [123]. Therefore, in most cases, rAAV viral titer measurements are unavailable during production. So, the challenge is to design a soft sensor that does not rely on rAAV viral titer measurements as its input. A soft sensor can have cell density or/and metabolite concentration (i.e., GLC, GLN, LAC, and AMM) as input and the rAAV viral titer only as output. For example, it is impossible to use past rAAV viral titer measurements to predict the present/future state because they are unavailable during the rAAV manufacturing process. In most cases, the quantification of viral titer in rAAV production is done only at the end of production. After the production is completed, the samples collected are used to quantify the viral titer, and the ddPCR takes 1 day to complete the process. Therefore, during the rAAV manufacturing, we have only cell density, GLC, GLN, LAC, and AMM that are measured offline every ∼30 min, or cell density that can be easily measured online.

**Possible Solution.** A soft sensor developed with a DDM, such as an LSTM, can learn how to predict the rAAV viral titer (CQA) based only on CPP, such as cell density, GLC, GLN, LAC, and AMM. More details about the development of an LSTM can be found in [92,124].

### 4.2. Multi-Step Forecasting

Time-series forecasting models predict future values of one or more dependent variables based on the past values of one or more independent variables [124,125]. Time series models can be univariate (with only one time-dependent or independent variable) or multivariate (with several time-dependent and independent variables). Although there are significant differences between univariate and multivariate models, most deep-learning models can handle both. For example, the model can have multivariate time series as inputs and univariate time series as the output [126,127]. Time series models can be defined with regard to the forecasting horizon in the n-step-ahead term. For example, if the model forecasts a point that is a one-time step ahead in the future, it is denoted as a one-step-ahead forecast. Similarly, multi-step forecast predicts *n* times-steps ahead [126,127]. In the majority of soft sensors applied in bioprocess applications, the one-step multivariate forecast is the most common approach used to estimate biomass and viral titer [49,86,128,129,130,131,132,133,134,135,136,137]. It is standard practice when forecasting the biomass to use lagged observations t−1 time instant as input variables to forecast the variables at the current time step *t*. An effective strategy for improving the process monitoring capability is to predict rAAV viral titer during the upstream process to assess future bioprocess conditions. It would be helpful to forecast the time of transfection and harvest (the time to stop the bioreactor after the production achieves the plateau). However, this would require a multivariate multi-step forecasting soft sensor, and the challenge here is to design a soft sensor to multi-step forecast the viral titer 24 or 48 h ahead. Unfortunately, this is difficult because of the accumulation of errors, reduced accuracy, and increased uncertainty.

**Figure 1:**
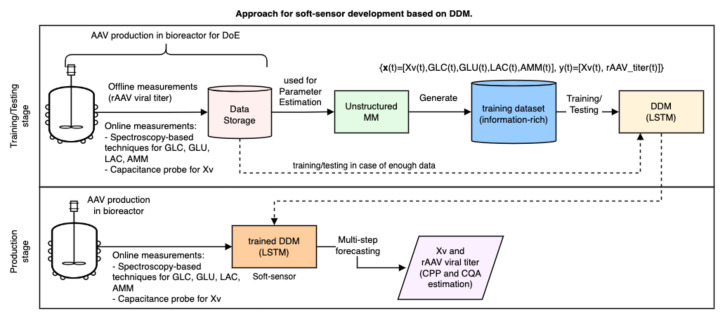
Soft sensor development approaches based on DDM for rAAV production monitoring. A possible approach is use unstructured MM to generate an information-rich training dataset and DDM for multi-step forecasting of rAAV viral titer.

**Possible Solution.** A soft sensor developed with a DDM, such as an LSTM, can be trained/tested to multi-step forecast a CQA based on previous time series data of CPPs [92]. LSTM is capable of capturing the patterns of long term, and this can provide greater accuracy. Furthermore, an ensemble approach could be used in LSTM development to perform forecasts with uncertainty [138]. Figure 1 shows a possible approach for data-driven soft sensor development for multi-step forecasting of rAAV viral titer. This approach aims to deploy unstructured MMs to generate training data sets for a DDM in order to learn to forecast (multi-step) CPP and CQA in rAAV production, including viable cell density (Xv) and rAAV viral titer, the two main critical process variables, see Figure 1. They are essential because the timing of transfection and harvest procedures, as well as monitoring the overall status of the culture, depends on the cell concentration. Furthermore, forecasting the rAAVviral titer is particularly interesting to the pharmaceutical manufacturing industry because it is a CQA. The first stage of this approach starts with the design of experiments (DoE) [116,139] to systematically investigate the relationship between CPPs (input factors such as Xv, GLC, GLN, LAC, and AMM) and rAAV viral titer (CQA) to have a better understanding of the impacts of different levels of each CPPs (different cell culture conditions) on the rAAV viral titer. In this stage, the measurements can be online for Xv, GLC, GLU, LAC, and AMM using spectroscopy-based techniques [64,118,119,120,121,122,140,141,142,143,144,145,146], and offline for rAAV viral titer using qPCR/ddPCR or ELISA, and they are stored in a data storage. These measurements from the DoE represent different cell culture conditions for rAAV production and are obtained with missing data and at different scales, since, for example, offline data are not collected at the same frequency as online data. Therefore, they should be integrated to generate an information-rich dataset (to enable effective DDM training), possibly performed with an unstructured MM. The measurements of data storage can be used for the parameter estimation of an unstructured MM regards the different rAAV production conditions, and it can generate a training dataset (information-rich) to enable DDMs such as LSTM models to learn to forecast the Xv and rAAV viral titer on different cell culture conditions of rAAV production. After the training, this DDM can be used in the production stage to perform a multi-step forecast of the timing of transfection and harvest procedures based on the CPP, and CQA estimation obtained from online measurements, see Figure 1. More details about the development of an LSTM can be found in [92,124].

This approach is based on [52,92,147]. It would not improve process comprehension regarding rAAV production, but it could help apply soft sensors in bioreactors for process optimization by efficiently forecasting a CQA, such as rAAV viral titer.

### 4.3. Multi-Phase Process

The upstream process of rAAV production is a multi-phase process composed of cell expansion and viral vector production. In the cell expansion phase, the cells are multiplied until they reach the required cell density to start the plasmid transfection, and in the viral vector production, the virus is produced for many days in the transiently transfected cells. Therefore, in developing a soft sensor methodology, it is essential to consider multi-phase characteristics of the upstream process in rAAV production since the relationships between state process variables (which can be physical, chemical, and biological) can vary substantially in the individual phases. Online monitoring should furthermore generate information regarding transitory process states [50]. Then, monitoring these phases can generate essential information that allows the tracking and fine-tuning of crucial process parameters in the upstream process, resulting in more efficient control, optimal harvest timing, and enhanced end-product quality.

**Possible Solution.** There are two potential directions. The first strategy would be to divide the process data sets into individual phase segments and develop separate sub-models for these segments [63]. In this approach, the overall model comprises one multi-variable sub-model for the cell expansion and the other for the viral vector production phase. A similar approach was developed by Selişteanu et al. [148] to model Monoclonal Antibody Production, where they created sub-models corresponding to the dynamics of viable cell concentration and dead cell concentration that are used in the time period of the exponential growth phase. A clear definition of the phases is a challenge in a multiple-model framework since biological effects can overlap [93]. However, algorithms for phase detection can be based on the shape of process trajectories or the correlation structure of process variables. For the detection and division of process phases, trajectory-based and correlation-based methods have been proposed in the literature [63]. The second approach involves modeling the entire upstream process rather than just one phase, considering interactions between phases or/and their relative relevance to the final product quality [149]. This method, known as multi-block, divides vast data collections by the process state variables into functional blocks, typically one block per phase. Then, the blocks are combined to most accurately forecast product state variables. An example of multi-block modeling is multi-block partial least squares [149].

### 4.4. Soft Sensor Development Composed of MMs

The soft sensor design based on HM, composed of MM and DMM, could be a challenging task in the case of rAAV production in bioreactors. The main concern is with MM, since the current state-of-the-art knowledge related to viral vector manufacturing modeling is still limited. There are relatively few works that focus on critical factors in rAAV manufacturing processes [53,110,111]. The first MM for rAAV production was published only recently by Nguyen et al. [110], and it is a structured MM. The model describes the kinetic behavior of transient transfection at the sub-cellular level and does not enable the macro-modeling of rAAV production at a bioreactor level (macro-scale) [116]. Furthermore, the 21 variables (species) available in this structured mechanistic model are not commonly measured in the bioreactor as bioprocess parameters, such as cell density, glucose (GLC), lactate (LAC), and oxygen [64]. This reduces the applicability of this structured MM to process optimization and the control of rAAV production at the bioreactor level. To our knowledge, there is currently no HM soft sensor developed and reported for rAAV production monitoring besides a proposal for HM development published recently by Joiner et al. [53]. The authors proposed a framework at a very abstract level that can be used as the initial direction for developing a hybrid mechanistic and data-driven model for rAAV production. However, this proposal suggests incorporating all mechanistic aspects of HEK293 metabolism and rAAV into a single HM, and this could be a very challenging development due to process complexity when involving structured and unstructured mechanistic models (multiscale modeling) [116] and a lack of information about the kinetic parameters in the literature.

**Possible solution.** One solution to facilitate the hybrid soft sensor development is to adapt unstructured MM from the literature since, most often, operational and control processes for optimal bioreactor performance can be derived from unstructured MM [150]. For example, unstructured MMs have been widely employed in monoclonal antibody production using Chinese hamster ovary (CHO) cells, and this approach could be used in rAAV production monitoring to speed up the optimization of the upstream process as well [2]. In this sense, the unstructured MM proposed by Kornecki et al. [84], which is also based on mammalian cell cultures in rAAV production, is a good starting point from which to design a hybrid soft sensor for rAAV production monitoring without considering the complexity of a triple plasmid transfection process. Therefore, this approach can help apply soft sensors in bioreactors for rAAV production monitoring, even though it cannot improve the understanding of the rAAV production mechanism.

**Figure 2:**
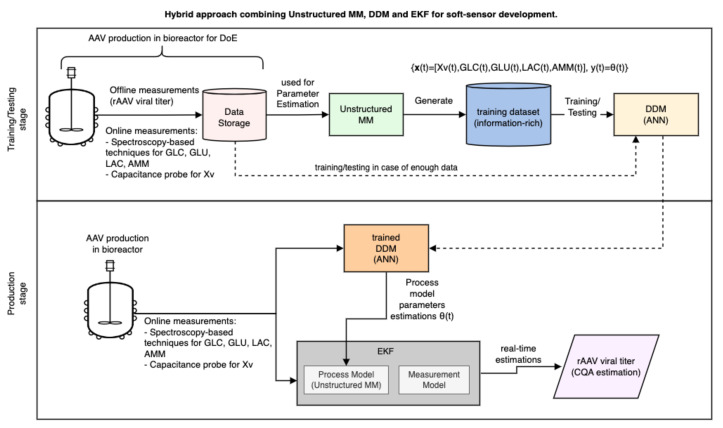
Soft sensor development approach based on HM for rAAV production monitoring. A possible approach is combining unstructured MM, DDM and EKF for real-time monitoring of rAAV viral titer.

Figure 2 shows a possible approach for developing a soft sensor based on HM, considering an unstructured MM as the sub-model. In this development approach, an HM for real-time monitoring of rAAV viral titer could be developed by combining an extended Kalman filter and an artificial neural network (ANN), see Figure 2. The EKF requires process and measurement models to estimate a process’s state variables and, generally, an unstructured MM is used as a process model. For example, the EKF proposed by Iglesias et al. [111] could reduce the number of devices for monitoring the state variables and enable the online monitoring of the rAAV viral titer. However, the parameter estimation for the process model (an unstructured MM) performed by the joint approach in EKF should be improved because it does not guarantee convergence. Alternatively, another method can estimate parameters outside the EKF calculation, such as an ANN. Therefore, the first stage of this approach would be training and testing an ANN to estimate the parameters of unstructured MM used as a process model of EKF, and this can be done similarly to the first stage of the strategy for soft sensor development based on DDM described in Section 4.2, see Figure 1. The difference is that here, the DDM (ANN) only estimates the parameters θ of the EKF process model. See Figure 2. After the training, this ANN model can be used in the production stage to work with the EKF to perform real-time estimations of rAAV viral titer using the online measurements of CPP such as Xv, GLC, LAC, and AMM; see Figure 2.

This approach is based on [111]. It would not improve process comprehension regarding rAAV production, but it might help apply soft sensors in bioreactors for process optimization by efficiently real-time monitoring a CQA, such as rAAV viral titer. It is important to point out that hybrid models involving Kalmen filter methods were proposed in [151,152] for the real-time monitoring and control of mammalian cell culture and presented significant results. More details about EKF can be found in [111].

## 5. Conclusions

Recombinant adeno-associated virus (rAAV) has rapidly emerged as one of the most attractive viral transfer tools, and several new advances have been made in different aspects of rAAV production. However, it is still a nascent gene therapy field that demands robust, scalable, and economically viable manufacturing processes. Therefore, optimizing upstream processing of rAAV production is extremely important to maximize viral titer and find stability for higher product quality, and soft sensors can be a valuable tool for this task. Furthermore, the cost-effectiveness of rAAV production is crucial for the commercialization of rAAV-based therapies, and soft sensing can play a key role in improving this cost-effectiveness by providing fast and low-cost monitoring of rAAV production. The others advantages of using soft sensors include productivity improvement, and product quality stabilization, which are the typical needs and goals of a manufacturing company. Despite the limited literature on the use of soft sensors in rAAV production, this review article discussed the advantages and challenges of applying soft sensors in this area and provided possible solutions to overcome these challenges. The challenges and requirements for the soft sensor development in upstream AAV production are: (i) Identifying the appropriate predictor variables (soft sensor inputs) without the use of rAAV viral titer; (ii) Design of a soft sensor for multi-step forecasting, including the time of transfection and harvest; (iii) Handling of the multi-phase characteristic of the upstream process; and (iv) Design of soft sensor composed of MMs. In the Biopharma 4.0 era, smart bio-manufacturing supported by soft sensors will produce widely accessible biopharmaceuticals that are safer, more effective, personalized, and cost-efficient. Additionally, sophisticated techniques, such as digital twins for the bioreactor process, may find a foundation in soft sensor technology.

## Data Availability

Not applicable.

## Supplementary Materials

The following are available at https://www.mdpi.com/article/10.3390/bioengineering10020229/s1, Table S1: Glossary.
