# Supplementary material for: rAAV Manufacturing: The Challenges of Soft Sensing during Upstream Processing
Source: Bioengineering (Basel). 2023 Feb 8;10(2):229. doi: 10.3390/bioengineering10020229 (PMC9951952; doi:10.3390/bioengineering10020229)
Supplement: Supplementary file 1 [file bioengineering-10-00229-s001.zip › bioengineering-2057621-supplementary.pdf]

Supplementary Materials: rAAV Manufacturing: the Challenges of Soft Sensing During Upstream Processing

Cristovão Freitas Iglesias Jr [2], Milica Ristovski [1,2], Miodrag Bolic [2], and Miroslava Cuperlovic-Culf [3,4,*]

1. Mechanistic Model Parameters Estimation

In order to characterize the dynamics of a biological system in an industrial bioprocess, mechanistic kinetic models have become more common, where development is performed in two steps [1,2]: The initial step is determining the MM type (structured or unstructured) and choosing the appropriate kinetic rate law based on available intracellular metabolism information. In addition, the system of ordinary differential equations-based dynamic models has to rely on kinetic parameters such as maximum reaction rates and kinetic rate constants to reflect the physiological and dynamic behaviour of the system. The last step is to assign appropriate numerical values to the MM parameters via parameter estimation. The parameter estimation of a system of ordinary differential equations (ODE) is a problem that necessitates finding the solution to a dynamic optimization problem, which is a non-convex problem that generally demands global optimization methods [1,2].

Analytic solutions, such as numerical methods, are common approaches for parameter estimation in biological ODE systems [3–5]. However, a new method called Neural Ordinary Differential Equation (NODE) is a particularly promising approach for learning latent dynamics of dynamical systems [6–9]. NODE naturally fits well as a latent-dynamics model in reduced-order modeling of physical processes because it learns the latent dynamics in the form of ODEs [6]. Moreover, it is a family of neural network models in which one or some hidden layers are implemented with an ODE solver [10–13]. NODE provides several advantages over standard numerical methods [6–12]. Firstly, the NODE solution is differentiable and is in closed analytic form. On the other hand, most other techniques offer a discretized solution or a solution with limited differentiability [7]. Secondly, the neural network-based method for solving a differential equation provides a solution with excellent generalizability characteristics [8,9]. Last, an important benefit of NODEs is the constant memory cost, where backward passes are computed using the adjoined sensitivity method rather than back-propagating through individual forward solver steps [10–12] The most important advantage of NODE for modeling a mechanistic kinetic model for rAAV production is its flexible learning even from irregularly sampled time-series data and its suitability to learning complex dynamical systems [8,9] making learning possible even from imperfect datasets generally only availe when measuring viral titer in rAAV production.

It is essential to point out that NODE enables getting the point estimates for the best parameters of ODE. However, data has noise, and in some cases, the fit should be generated with some uncertainty, i.e., uncertainty quantification [14,15]. This could be done with Bayesian inference enabling parameter estimation with quantified uncertainty that can be represented by mean ± Standard Deviation (StD). Bayesian inference provides a robust approach to parameter estimation with quantified uncertainty using a posterior distribution [14,15].

2. Glossary

Table S1. Glossary.

Term	Description
Adeno-associated viruses (AAVs)	a non-enveloped virus that can be engineered to deliver DNA to target cells.
Antigen-presenting cells (APCs)	a heterogeneous group of immune cells that mediate the cellular immune response by processing and presenting antigens for recognition by certain lymphocytes such as T cells.
Assembly-activating protein (AAP)	a non-structural protein expressed from a non-canonical CTG start codon of an overlapping reading frame embedded within the cap gene.
Autoendocoder (AE)	a specific kind of unsupervised artificial neural network that provides compression and other functionality in the field of machine learning.
Bayesian inference	a method of statistical inference in which Bayes' theorem is used to update the probability for a hypothesis as more evidence or information becomes available.
Biopharma 4.0	a hybrid approach as it seeks to integrate old technologies such as continuous processing, automation, and process analytics, with newer approaches such as digitalization, advanced data management, and predictive modeling.
Bioreactors	an engineered system, deployed to facilitate the growth of biological mass through the transformation or degradation of material fed to the reactor.
Biomanufacturing	manufacture of products using living systems such as microorganisms, animal cells, or plant cells.
Capsid protein (Virus)	a structural element of the virion responsible for encapsidation of the viral genome and nucleocapsid assembly.
Cell expansion	a process where cells (e.g. adherent HEK293T or suspension HEK293) are expanded to a desired cell density.
Convolutional Neural	

Continued on next page

Table S1 – continued from previous page

Term	Description
Network (CNN)	a network architecture for deep learning which learns directly from data, eliminating the need for manual feature extraction.
Critical quality attribute (CQA)	a physical, chemical, biological, or microbiological property that, to ensure the desired product quality of a pharmaceutical or biopharmaceutical product, must fall within a defined limit, range, or distribution.
Data-Driven Model (DDM)	a model built purely on empirical observations of a process.
Droplet Digital PCR (ddPCR)	a method for performing digital PCR that is based on water-oil emulsion droplet technology.
Deep Learning (DL)	a subset of the machine learning that allows machines to solve complex problems from the multidimensional, complex datasets.
Dielectric spectroscopy (DS)	a method frequently used to study the response of a sample subjected to an applied electric field of fixed or changing frequency.
Differential digital holographic microscopy (DDHM)	a digital holography applied to microscopy.
Digital twins	a dynamic virtual copy of a physical asset, process, system or environment that looks like and behaves identically to its real-world counterpart.
Enzyme-linked immunosorbent assay (ELISA)	a plate-based assay technique designed for detecting and quantifying soluble substances such as peptides, proteins, antibodies, and hormones.
Extended Kalman Filter (EKF)	a method that handles nonlinear process and measurement models by resorting to linearization for the propagation of error covariance matrix and Kalman gain computation.
Food and Drug Administration (FDA)	agency of the US Department of Health and Human Services that is responsible for protecting public health by regulating the manufacturing, marketing, and distribution of drugs, biological products, food, cosmetics, and radiation-emitting products.

Continued on next page

Table S1 – continued from previous page

Term	Description
Gaussian Processes Regression (GPR)	a nonparametric, Bayesian approach to solve regression and probabilistic classification problems.
Helper plasmid (pHelper)	a plasmid that contains required E2A, E4, and VA RNA adenoviral genes.
High-performance liquid chromatography (HPLC)	a technique in analytical chemistry used to separate, identify, and quantify each component in a mixture.
Hybrid Model (HM)	a new paradigm in modeling that combines the interpretability, robust foundation and understanding of a physics-based approach with the accuracy, efficiency, and automatic pattern-identification capabilities of advanced data-driven machine learning and artificial intelligence algorithms to produce less uncertain results.
Key Performance Indicator (KPI)	a metric for the status of each step in a pharmaceutical manufacturing process.
Long Short Term Memory (LSTM)	a type of recurrent neural network capable of learning order dependence in sequence prediction problems.
Mechanistic Model (MM)	a mathematical description of the elements forming a system, their mutual interactions and the interaction with the environment.
Multi-step forecasting	task of predicting a sequence of future values using only the values observed in the past.
Near-infrared spectroscopy (NIRS)	a noninvasive technology that continuously monitors regional tissue oxygenation.
Neural Ordinary Differential Equation (NODE)	a family of neural network models in which one or some hidden layers are implemented with an ordinary differential equation solver.
Neutralizing antibodies (NAbs)	an antibody that defends a cell from a pathogen or infectious particle by neutralizing any effect it has biologically.
Ordinary differential equation (ODE)	an equation that involves some ordinary derivatives (as opposed to partial derivatives) of a function.

Continued on next page

Table S1 – continued from previous page

Term	Description
packaging plasmid (pRC)	a plasmid that contains contains the structural (gag), and replication (pol) genes which code for some of the proteins required to produce the lentivirus.
Parameter estimation	an experimental determination of values of parameters that govern the system behavior, assuming that the structure of the process is known.
Partial Least Squares (PLS)	a technique that reduces the predictors to a smaller set of uncorrelated components and performs least squares regression on these components, instead of on the original data.
Plasmid development	a process where a cis-plasmid (that encodes a gene of interest (GOI) flanked by the AAV inverted terminal repeats (ITRs)), a trans-plasmid (that encodes the AAV rep and cap genes), and a helper plasmid (that encodes adenovirus (Ad) helper genes, E2A, E4, and VA RNA) are designed and produced.
Plasmid Transfection	a process where plasmids are introduced into cells after it has reached a desired cell density.
PCR	an in vitro method for making many copies of a DNA region.
Process analytical technology (PAT)	the process of ensuring that final product quality meets specifications by designing, analyzing, and controlling manufacturing though periodic and/ or continuous measurement of critical quality and performance attributes.
quantitative Polymerase Chain Reaction (qPCR)	a technology used for measuring DNA using PCR.
Quality by design (QbD)	a systematic approach to development that applies sound science, process and product understanding, process control, and quality risk management to ensure the predefined product and process objectives are met.
Recurrent Neural Network (RNN)	a type of artificial neural network which uses sequential data or time series data.

Continued on next page

Table S1 – continued from previous page

Term	Description
Small ubiquitin-like modifier (SUMO)	a member of the superfamily of ubiquitin-like polypeptides that become covalently attached to various intracellular target proteins as a way to alter their function, location, and/or half-life.
Soft-sensors	a combination of process data (input) and a model that uses these input data to predict a target quantity (output).
Support Vector Machine (SVM)	a popular Supervised Learning algorithms, which is used for Classification as well as Regression problems.
Time-series	a series of data points indexed (or listed or graphed) in time order.
Upstream process	the first step of bioprocess from early cell isolation and cultivation, to cell banking and culture development of the cells until final harvest where the desired quantity is reached.
Viable cell density (VCD)	a key performance indicator (KPI) of the impact of critical process parameters on the operational performance of the culture.
Viral vector production	a process where transiently transfected cells are then allowed to produce the virus for several days.

3. References

1. Dua, V.; Dua, P. A simultaneous approach for parameter estimation of a system of ordinary differential equations, using artificial neural network approximation. *Industrial & engineering chemistry research* **2012**, *51*, 1809–1814.
2. Kyriakopoulos, S.; Ang, K.S.; Lakshmanan, M.; Huang, Z.; Yoon, S.; Gunawan, R.; Lee, D.Y. Kinetic modeling of mammalian cell culture bioprocessing: the quest to advance biomanufacturing. *Biotechnology Journal* **2018**, *13*, 1700229.
3. Petre, E.; Selişteanu, D. Model approximation and simulations of a class of nonlinear propagation bioprocesses. *Numerical Analysis-Theory and Application* **2011**, pp. 211–230.
4. Dürr, R.; Waldherr, S. Hybrid simulation algorithm for efficient numerical solution of population balance equations. *IFAC-PapersOnLine* **2018**, *51*, 290–295.
5. Xu, P. Analytical solution for a hybrid Logistic-Monod cell growth model in batch and continuous stirred tank reactor culture. *Biotechnology and bioengineering* **2020**, *117*, 873–878.
6. Lee, K.; Parish, E.J. Parameterized neural ordinary differential equations: Applications to computational physics problems. *Proceedings of the Royal Society A* **2021**, *477*, 20210162.
7. Okereke, R.N.; Maliki, O.S.; Oruh, B.I. A novel method for solving ordinary differential equations with artificial neural networks. *Applied Mathematics* **2021**, *12*, 900–918.
8. Yang, Y.; Hou, M.; Luo, J. A novel improved extreme learning machine algorithm in solving ordinary differential equations by Legendre neural network methods. *Advances in Difference Equations* **2018**, *2018*, 1–24.
9. Bradley, W.; Boukouvala, F. Two-Stage Approach to Parameter Estimation of Differential Equations Using Neural ODEs. *Industrial & Engineering Chemistry Research* **2021**, *60*, 16330–16344.
10. Rackauckas, C.; Innes, M.; Ma, Y.; Bettencourt, J.; White, L.; Dixit, V. Diffeqflux. jl-A julia library for neural differential equations. *arXiv preprint arXiv:1902.02376* **2019**.
11. Xia, H.; Suliafu, V.; Ji, H.; Nguyen, T.; Bertozzi, A.; Osher, S.; Wang, B. Heavy ball neural ordinary differential equations. *Advances in Neural Information Processing Systems* **2021**, *34*.
12. Chen, R.T.; Rubanova, Y.; Bettencourt, J.; Duvenaud, D. Neural ordinary differential equations. *arXiv preprint arXiv:1806.07366* **2018**.
13. Dhadphale, J.M.; Unni, V.R.; Saha, A.; Sujith, R. Neural ODE to model and prognose thermoacoustic instability. *Chaos: An Interdisciplinary Journal of Nonlinear Science* **2022**, *32*, 013131.
14. Alahmadi, A.A.; Flegg, J.A.; Cochrane, D.G.; Drovandi, C.C.; Keith, J.M. A comparison of approximate versus exact techniques for Bayesian parameter inference in nonlinear ordinary differential equation models. *Royal Society open science* **2020**, *7*, 191315.
15. Ge, H.; Xu, K.; Ghahramani, Z. Turing: a language for flexible probabilistic inference. In Proceedings of the International conference on artificial intelligence and statistics. PMLR, 2018, pp. 1682–1690.
